# *Mycobacterium tuberculosis* Uses Mce Proteins to Interfere With Host Cell Signaling

**DOI:** 10.3389/fmolb.2019.00149

**Published:** 2020-01-08

**Authors:** Katherine Fenn, Chi Tung Wong, Vidya Chandran Darbari

**Affiliations:** School of Biological and Chemical Sciences, Queen Mary University of London, London, United Kingdom

**Keywords:** Mce protein, host cell signaling, host-pathogen interaction, *Mycobacterium tuberculosis*, ERK, EF1A1, integrin

## Abstract

Tuberculosis continues to be the main cause for mortality by an infectious agent, making *Mycobacterium tuberculosis one* of the most successful pathogens to survive for long durations within human cells. In order to survive against host defenses, *M. tuberculosis* modulates host cell signaling. It employs many proteins to achieve this and the Mce proteins are emerging as one group that play a role in host cell signaling in addition to their primary role as lipid/sterol transporters. Mce proteins belong to the conserved Mce/MlaD superfamily ubiquitous in diderm bacteria and chloroplasts. In mycobacteria, *mce* operons, encode for six different Mce proteins that assemble with inner membrane permeases into complexes that span across the mycobacterial cell wall. Their involvement in signaling modulation is varied and they have been shown to bind ERK1/2 to alter host cytokine expression; eEF1A1 to promote host cell proliferation and integrins for host cell adherence and entry. Recently, structures of prokaryotic Mce/MlaD proteins have been determined, giving an insight into the conserved domain. In this mini-review, we discuss current evidence for the role of mycobacterial Mce proteins in host cell signaling and structural characteristics of the protein-protein interactions coordinated by the human proteins to modulate the host signaling.

## Introduction

The intracellular pathogen, *Mycobacterium tuberculosis*, has sophisticated mechanisms to promote its own survival within humans. The tussle of bacterial survival vs. disease progression is governed by dynamic interactions between *M. tuberculosis* and host cells. Inhaled aerosolized bacteria primarily encounter alveolar macrophages and their ability to persist in an antimicrobial environment is critical for establishing an infection (Simmons et al., 2018). One of the intriguing methods of survival is how the pathogen modulates host cell signaling for its own advantage (Ferraris et al., 2018). There are several examples of this strategy including PtpA binding of host ubiquitin leading to the suppression of host Jnk, p38, and NF-κB pathways (Wang et al., 2015); ESAT-6 binding to host TLR2 leading to inhibition of NF-κB activation within host cells (Pathak et al., 2007); Zmp1 prevents host phagosome maturation by inhibiting the production of IL-1β (Master et al., 2008); and lipoarabinomannan (LAM) prevents apoptosis of host cells via promoting phosphorylation of Bad in the PI-3K/Akt pathway to name a few (Maiti et al., 2001).

**Figure 1:**
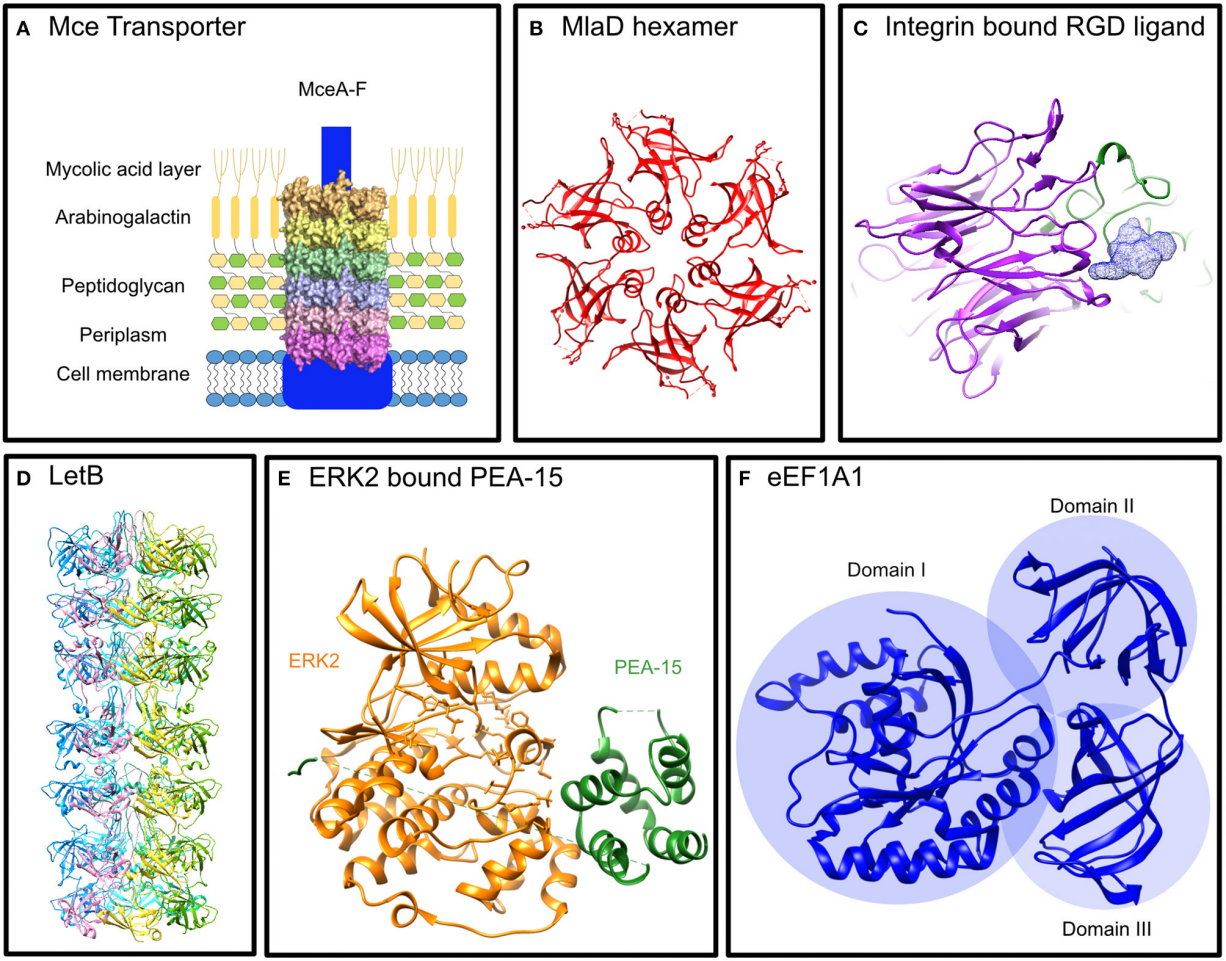
Structures of Mce proteins from *E. coli* and structures of proteins known to interact with the *M. tuberculosis* Mce proteins. **(A)** Mce Transporter: Proposed structure of an Mce Transporter that sits in the mycobacterial membrane interacting with lipids and signaling proteins. **(B)** MlaD hexamer: The *E. coli* Mce protein, MlaD, forms a hexamer of the ubiquitous Mce domain (PDB: 5UW2) (Ekiert et al., 2017). **(C)** Integrin bound RGD Ligand: The propeller of the α subunit and the βA domain of the ß subunit binds the RGD motif of its ligand to initiate intracellular signaling (Xiong et al., 2002) (PDB: 1L5G). **(D)** LetB: An *E. coli* protein with seven tandem Mce domains that forms a hexamer that spans the periplasm (Isom et al., 2019) (PDB available from http://bhabhaekiertlab.org/pdb-links). **(E)** ERK2 bound PEA-15: PEA-15 binds ERK2 in a bipartite manner to inhibit it (Mace et al., 2013) (PDB: 4IZ5). **(F)** eEF1A1: The elongation factor alpha 1 has three distinct domains, I, II, III (Andersen et al., 2000) (PDB: 1F60).

**Figure 2:**
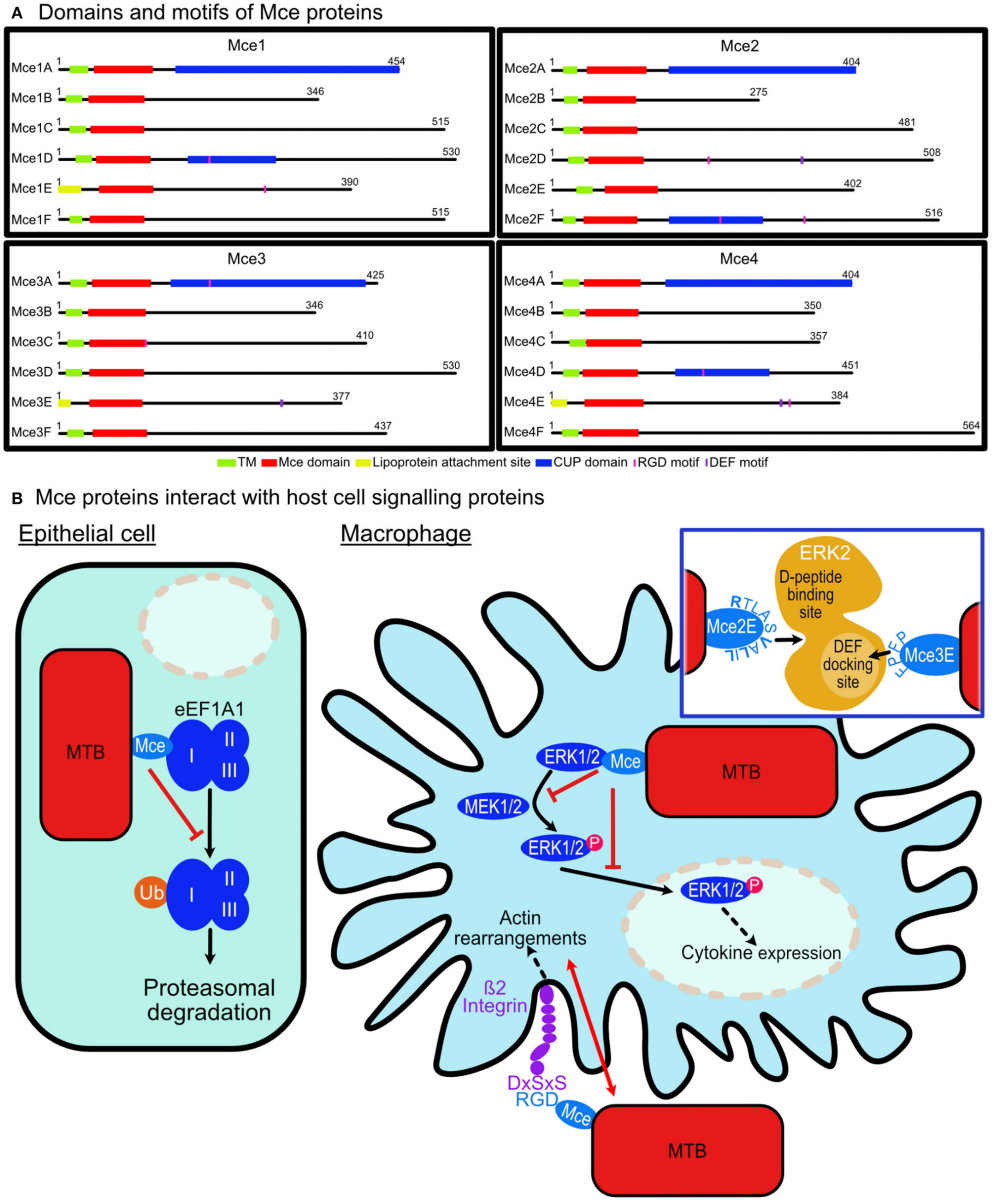
*M. tuberculosis* Mce proteins are involved in modulating host cell signaling. **(A)** Domains and motifs of Mce proteins: Each *M. tuberculosis* Mce (1–4) Transporter is composed of six Mce proteins (A–F). Twenty-one have transmembrane domains (TM) and the remaining three (1E, 3E, 4E) contain lipoprotein attachment sites. Each protein has a conserved Mce domain with unique C-terminal domains including the Cholesterol uptake porter (CUP) domain (1A, 1D, 2A, 2F, 3A, 4A, 4D); RGD motifs for probable integrin binding (1D, 1E, 2D, 2E, 3A, 3C, 4D, 4E); DEF motifs (2D, 3E, 4E). **(B)** Mce proteins interact with host cell signaling proteins. *Epithelial cell:* Mce2E of *M. tuberculosis* binds to domain I of eEF1A1 preventing its K-48 linked ubiquitination and preventing its proteasomal degradation leading to epithelial cell proliferation. *Macrophage cell:* Mce3C from *M. tuberculosis* binds ß2 integrin on macrophage surfaces thereby co-ordinating actin rearrangements to enable its cell entry. Mce3C binds ß2 integrin via an RGD motif interaction with DxSxS motif on the integrin. Once intracellular, Mce2E and Mce3E bind to ERK1/2 preventing its phosphorylation by MEK1/2 and its nuclear translocation leading to suppression of the activation of the ERK signaling pathway. *Inset:* Mce3E binds using a D- domain motif binding to a site away from the activation loop of ERK. Mce2E binds using a DEF-motif, FPFP, binding to the DEF binding site located at the activation loop of ERK.

The role of Mce (mammalian cell entry) protein assemblies from *M. tuberculosis* in host cell signaling modulation is starting to emerge. The pathogen contains four homologous *mce* operons that each encode six Mce proteins [Mce(1-4)A-F] and two permease proteins which assemble with an ATPase, termed MceG, to form assemblies resembling ABC-transporters that reside in the extracellular membrane (Figure 1A). These assemblies are proposed to be lipid or steroid transporters that allow the pathogen to uptake nutrients in antimicrobial environments (Casali and Riley, 2007). The Mce proteins are integral membrane proteins and some are predicted to be surface exposed. All 24 Mce proteins have the conserved Mce domain, with unique C-terminal domains including the Cholesterol uptake porter domain (1A, 1D, 2A, 2F, 3A, 4A, 4D); RGD motifs for probable integrin binding (1D, 1E, 2D, 2E, 3A, 3C, 4D, 4E); DEF motifs (2D, 3E, 4E) [Schematic representation of Mce(1-4)A-F proteins in Figure 2A].

Each Mce protein contains a single homologous Mce domain that is ubiquitous across double-membrane bacteria and eukaryotic chloroplasts (Casali and Riley, 2007; Ekiert et al., 2017). The proteins have been linked to lipid homeostasis or cell adhesion across these organisms. The best characterized are prokaryotic Mce proteins including MlaD and LetB from *Escherichia coli* (Ekiert et al., 2017; Isom et al., 2019). MlaD (Maintenance of Lipid Asymmetry) has a single Mce domain that forms a homo-hexameric ring (Figure 1B) and resides in the periplasm tethered to the inner membrane as part of an ABC-transporter complex (Ekiert et al., 2017). Similarly, LetB (or YebT) with its seven tandem Mce domains forms a hexameric assembly tethered to the inner membrane that spans the periplasm (Figure 1D; Isom et al., 2019). The Mce domains adopt a seven-stranded beta barrel fold (Ekiert et al., 2017). The *E. coli* Mce proteins are all located in the periplasm whereas some mycobacterial Mce proteins are extracellular allowing for potential functions beyond transport. In fact, MAM7 (multivalent adhesion factor 7), a gram-negative outer membrane Mce protein with seven tandem Mce domains from *Vibrio parahaemolyticus* has been shown to bind both host lipids and proteins to enable host cell adhesion alluding to the role of Mce proteins in manipulating host cell signaling (Lim et al., 2014).

In this minireview, the current understanding of *M. tuberculosis* Mce proteins and their roles in manipulating host cell signaling is examined.

## Mitogen-Activated Protein Kinase (MAPK) Signaling

*Mycobacterium tuberculosis* evades detection by the host immune system by interfering with cytokine production (Domingo-Gonzalez et al., 2016) and Mce proteins have been implicated in this. Mce3E has been demonstrated to downregulate host cytokine expression and aid the survival of *M. tuberculosis* once it has entered macrophages by inhibiting the activation of the extracellular signal-regulated kinase 1/2 (ERK1/2) signaling pathway (Li et al., 2015). Mce3E appears to bind to ERK1/2 via its DEF motif. This binding prevents ERK1/2 phosphorylation by MEK1 in the cytoplasm and blocks the nuclear translocation of p-ERK1/2 thereby preventing the activation of the ERK1/2 signaling pathway. The ultimate response is the suppression of TNF and IL-6 cytokine expression (Figure 2B). Previous studies indicate that the ERK docking motif has a consensus sequence of FXFP (Jacobs et al., 1999; Fantz et al., 2001; Galanis et al., 2001; Fernandes and Allbritton, 2009; Upadhyay et al., 2018) and in Mce3E it is FPFP. The first phenylalanine residue is the most critical reside in the sequence of Mce3E as mutating it abolishes ERK binding *in vitro* (Li et al., 2015).

A more recent finding by Qiang et al. shows that Mce2E suppresses macrophage immune responses (Qiang et al., 2019). It inhibits production of cytokines TNF and IL-6 by suppressing the activation of the ERK and JNK pathways. Mce2E, like Mce3E, blocks the interactions of ERK1/2 and JNK with their upstream kinases, MEK1 and MKK4, respectively. Mce2E achieves this through a non-canonical D motif represented by (K/R)1-2-(X)2-6-ØA-X-ØB (where ØA and ØB are hydrophobic residues Leu, Ile, or Val; Figure 2). Mutations of V173, L175, and L177 from the D motif of Mce2E to alanine residues prevented its binding to ERK or JNK (Qiang et al., 2019).

Structural analysis of ERK2 with a regulatory protein, PEA-15 (15 kDa phosphoprotein enriched in astrocytes), reveals a binding interaction that might be employed by Mce2E and Mce3E. PEA-15 binds to ERK1/2 preventing its nuclear translocation similar to the mechanism exhibited by Mce2E and Mce3E. ERK2 consists of two domains: a smaller N-terminal domain that consists of mainly -strands and sits on the C-terminal domain that is comprised of mostly alpha helices. The C-terminal domain contains the activation loop which is dually phosphorylated for activation (Figure 1E). PEA-15 binds in a bipartite manner using a DEF-motif to bind directly into activation loop of ERK2 and a C-terminal D-domain to bind the D-peptide docking site located on the opposite side of the C-terminal domain (Figure 1E; Mace et al., 2013). This structural insight provides clues to the interactions of Mce proteins with ERK1/2. Mce3E binds to ERK1/2 with the DEF-motif and binds within the activation loop whilst Mce2E binds using the D-domain at a site on the opposite side to the activation loop (Figure 2B). It is interesting that both proteins bind ERK1/2 but using different binding interactions to bind different regions of the protein.

## Proteasomal Degradation Pathways

In addition to its suppression of macrophage immune responses, Mce2E has been shown to promote epithelial cell proliferation (Qiang et al., 2019). This was in an Mce2E D-motif independent manner and indicates the scope for interactions with other proteins in additional signaling pathways. Eukaryotic translation elongation factor 1 alpha 1 (eEF1A1), a conserved GTP-binding protein and the second most abundant protein after actin, was identified as such a protein (Mateyak and Kinzy, 2010). Mce2E was shown to prevent its proteasomal degradation by suppressing its K48-linked polyubiquitination. Mce3E does not exhibit the same cell proliferation function in epithelial cells (Qiang et al., 2019). *M. tuberculosis* disrupting host cell degradation pathways has been documented previously with the PtpA protein (Wang et al., 2015) and its link to the Mce domain expands its function to preventing host protein degradation.

eEF1A1 is expressed in the majority of cell types and in its primary function when GTP-bound, it binds aminoacylated-tRNA delivering it to the A site of ribosomes during polypeptide chain elongation in translation (Mateyak and Kinzy, 2010). It is comprised of three domains: I, II, and III (Figure 1F). The largest domain, domain I, consists a Rossmann fold topology and binds GTP/GDP (Abbas et al., 2015). Domain II and III each form a ß-barrel with domain II sitting on top of domain III. Domain I and II bind eEF1B for nucleotide exchange whilst domain II and III binds aa-tRNAs (Andersen et al., 2001). Mce2E binds to domain I and the binding is attributed to the Mce domain region of Mce2E (Figure 2B; Qiang et al., 2019). The exact binding residues or region of either domain have not been elucidated nor has it been determined if the GTP/GDP binding state of eEF1A1 is critical for binding.

Beyond the canonical role of eEF1A1 in protein translation, it has several non-canonical roles including proteolysis, protein nuclear export, cytoskeleton organization, and apoptosis (Mateyak and Kinzy, 2010). Whether the binding of eEF1A1 has any tangible effects on these pathways remains to be seen. Equally whether the binding is observed in other cell types or is only observed in epithelial cells is an intriguing question. The use of eEF1A1 for pathogenesis is well-known and its multiplicity of functions is utilized by several viruses including HIV-1, West Nile Virus and HPV (Li et al., 2013).

## Integrin Signaling

Pathogens are known to exploit receptor-mediated signaling to avoid antimicrobial strategies of host cells including to prevent or abolish phagocytosis. *M. tuberculosis* appears to employ receptor binding as another method to utilize host signaling for its advantage. Zhang et al. demonstrated that Mce3C, present on the surface of mycobacteria, binds to ß2 integrin promoting adherence and entry into macrophages. The binding is RGD motif dependent and leads to integrin dependent SFKs-Syk-Vav-Rho-ROCK signal transduction inducing local actin rearrangements thus promoting mycobacterial entry. The RGD motif of Mce3C has a high affinity for the leucocyte-restricted ß2 integrin binding to its I-like domain (Figure 2B). Mutation of the RGD motif to RAA abolished the interaction between Mce3C and ß2 integrin blocking Mce3C association to macrophages. Equally mutation of the I-like domain conserved motif DxSxS to AxAxA abolished this association (Zhang et al., 2018).

The RGD motif is a well-documented ligand binding motif of integrins (Figure 1C) and one-third of integrins have been shown to bind it (Xiong et al., 2002; Barczyk et al., 2010). Integrins are membrane proteins with large extracellular regions comprised of heterodimers of α and β subunits. A structural analysis of the binding by integrins to the RGD motif shows that the ligand binds at the interface between the propeller of the α subunit and the βA domain of the ß subunit (Xiong et al., 2002). Several microbial proteins have been shown to bind integrins via an RGD-motif (Ruoslahti, 1996) but perhaps the most interesting to note here is the Mce protein from *Leptospira interrogans* strain Lai. It binds to α5ß1, α_v_ß3, and ß2 integrins to promote pathogen adhesion and invasiveness (Zhang et al., 2012; Cosate et al., 2016).

Of the 24 Mce proteins in *M. tuberculosis*, eight contain RGD motifs with a total of 10 (Mce1E and Mce2E contain two each; Figure 2A). There are two proteins in each Mce assembly that contains the sequence. The presence of them and the confirmation that other Mce proteins bind integrins suggests that the Mce assemblies have a role in integrin binding to enable their adhesion and entry into host cells. Several questions remain including are all these sequences accessible to the receptors? Do they bind different integrins to allow for entry into different cells? Do they bind different integrins to modulate different signaling pathways?

## Conclusion and Future Perspectives

The binding of Mce proteins from *M. tuberculosis* to different host signaling proteins highlights their role beyond lipid transport. Thus far, they have been implicated in altering cytokine expression through interaction with ERK1/2; promoting cell proliferation through preventing proteasomal degradation of eEF1A1 and enabling cell adherence and entry through interactions with integrins. The current research is limited to a few studies encompassing three out of the 24 Mce proteins in *M. tuberculosis*. When all 24 are considered in the context of their membrane complexes their effect on host cell signaling could be vast and varied. There is some evidence to suggest that the four *mce* operons are expressed at different times during the infection life-cycle (Joshi et al., 2006; Casali and Riley, 2007; Klepp et al., 2012), however this information is inadequate to tease out the contributions of the individual Mce complexes to host-pathogen interactions for their contribution in sustaining infection. The differential expression can have an impact on which host signaling pathways are manipulated at a particular period of infection depending on the pathogens requirements. However, further studies are required to elucidate when and how each transporter is utilized by the mycobacteria. Equally how the Mce proteins interact with host proteins when assembled in complexes on the mycobacterial membrane remains unclear and the previous studies have all related to single expressed Mce proteins rather than whole operons expressed as complexes. Mce2E has been shown to have dual functions and the intriguing question is whether this dual binding is displayed in every cell type. Moreover, how many of the remaining Mce proteins will have multiple interaction partners in host cells and how will these interactions connect to their other functions as lipid/steroid transporters affecting membrane integrity. The understanding of the interplay between multiple *M. tuberculosis* proteins such as Mce, ZmpI, and PtpA interacting with many signaling pathways is key to understanding the sophistication the pathogen employs to survive.

## Author Contributions

KF and VD conceptualized the manuscript. KF, CW, and VD wrote the manuscript.

### Conflict of Interest

The authors declare that the research was conducted in the absence of any commercial or financial relationships that could be construed as a potential conflict of interest.
